# *Crataegus laevigata* Suppresses LPS-Induced Oxidative Stress during Inflammatory Response in Human Keratinocytes by Regulating the MAPKs/AP-1, NFκB, and NFAT Signaling Pathways

**DOI:** 10.3390/molecules26040869

**Published:** 2021-02-06

**Authors:** Quynh T. N. Nguyen, Minzhe Fang, Mengyang Zhang, Nhung Quynh Do, Minseon Kim, Sheng Dao Zheng, Eunson Hwang, Tae Hoo Yi

**Affiliations:** College of Life Sciences, Kyung Hee University, 1732, Deogyeong-daero, Giheung-gu, Yongin-si, Gyeonggi-do 17104, Korea; quynhnguyen@khu.ac.kr (Q.T.N.N.); mincheolbang1030@gmail.com (M.F.); zhangmy@khu.ac.kr (M.Z.); quynhnhung96@khu.ac.kr (N.Q.D.); dbs03067@khu.ac.kr (M.K.); sdjeong0719@khu.ac.kr (S.D.Z.)

**Keywords:** *Crataegus laevigata*, oxidative stress, inflammation, keratinocytes, LPS, MAPK/AP-1, NF-κB, NFAT

## Abstract

*Crataegus laevigata* belongs to the family Rosaceae, which has been widely investigated for pharmacological effects on the circulatory and digestive systems. However, there is limited understanding about its anti-oxidative stress and anti-inflammatory effects on skin. In this study, 70% ethanol *C. laevigata* berry extract (CLE) was investigated on lipopolysaccharide (LPS)-stimulated keratinocytes. The LPS-induced overproduction of reactive oxygen species (ROS) was suppressed by the treatment with CLE. In response to ROS induction, the overexpression of inflammatory regulating signaling molecules including mitogen-activated protein kinases (MAPK)/activator protein-1 (AP-1), nuclear factor kappa-light-chain-enhancer of activated B cell (NF-κB), and nuclear factor of activated T-cells (NFAT) were reduced in CLE-treated human keratinocytes. Consequently, CLE significantly suppressed the mRNA levels of pro-inflammatory chemokines and interleukins in LPS-stimulated cells. Our results indicated that CLE has protective effects against LPS-induced injury in an in vitro model and is a potential alternative agent for inflammatory treatment.

## 1. Introduction

Inflammation and oxidative stress are unavoidable by-products of normal cellular activities; however, excessive oxidative stress and inflammation can cause acute and chronic diseases, such as urticaria, eczema, psoriasis, and atopic dermatitis, and accelerate the aging process [1,2,3,4,5]. During the inflammatory process, excessive production of reactive oxygen species (ROS) results in oxidative stress [6,7,8]. Exposure to an inflammatory component of gram-negative bacteria, lipopolysaccharide (LPS), leads to ROS-mediated activation of cell inflammatory signaling pathways such as mitogen-activated protein kinase/activator protein 1 (MAPK)/AP-1 and nuclear factor kappa-light-chain-enhancer of activated B cells (NF-κB) [9,10,11]. At the transcription level, MAPK phosphorylation, AP-1 activation, and nuclear localization of NF-κB regulate mRNA expression of key inflammatory mediators including CC chemokines such as thymus- and activation-regulated chemokine/CC chemokine ligand 17 (TARC/CCL17), macrophage-derived chemokine/CC chemokine ligand 22 (MDC/CCL22), regulated on activation normal T expressed, and secreted/CC chemokine ligand 15 (RANTES/CCL5) and interleukin-8 (IL-8) [12,13,14,15,16]. Therefore, agents that decrease ROS formation and inhibit inflammatory mediator release via the stated signaling pathways are potential candidates for treatment of skin inflammation.

Although the inflammatory process is generally controlled by evolutionarily conserved signaling pathways such as MAPK and NF-κB, increasing evidence demonstrates that factors that more recently emerged in evolution modulate particular features of inflammatory responses. In particular, the nuclear factor of activated T cells (NFAT), which is evolutionarily related to the transcription factors of the NF-κB family, is an essential regulator of expression of potent immunomodulatory cytokines [17,18,19]. Additionally, it was shown that NFAT phosphorylation state and its nuclear translocation are principally regulated by enzyme P38 of the MAPK family. Moreover, NFAT proteins are found not only in T cells and immune cells, but also in other sites of the body including muscle, bone, neurons, and skin tissue. Under stimulation by inflammatory inducers such as LPS, NFAT promotes gene transcription of TNF-α, COX-2, and NOS2, which are associated with inflammation. The NFAT-targeting agent, tacrolimus, is a well-known alternative for topical corticosteroids (e.g., dexamethasone, hydrocortisone), used for treatment of redness, itching, and inflammation on sensitive skin areas [20]. However, like some corticosteroids, prolonged usage of tacrolimus has shown several side effects such as skin flushing, soreness, and increased blood pressure [20]. Thus, alternative agents should be developed to ensure safe treatment.

*Crataegus laevigata*, also known as midland hawthorn, belongs to the family Rosaceae, being widely distributed in western and central Europe, and is well-known for its pharmacological effect on cardiovascular disease [21,22,23,24,25]. The phytoactive secondary compounds dominantly present in hawthorn fruits are proanthocyanidins (polymers of (epi)catechin units) [26,27] and flavonoids [28], which have been investigated for various pharmacological effects, especially anti-inflammation [25,28,29,30]. Nevertheless, contrary to the well-investigated cardiovascular effects, data about other pharmacological activities of *C. laevigata* extracts, particularly those on anti-inflammatory effects on skin diseases, have been poorly studied. In this study, the promising inhibitory effects of *C. laevigata* fruit extract (CLE), which is rich in chlorogenic acid and (−)-epicatechin, on LPS-stimulated ROS formation, synthesis of pro-inflammatory molecules, and underlying signal transduction pathways, were investigated in human dermal keratinocytes. We demonstrated that, under LPS-stimulated oxidative stress, CLE alleviates ROS production and pro-inflammatory mediator release by inhibiting MAPK/AP-1 and NF-κB pathways. For the first time, the results revealed that CLE can regulate innate immune defenses through NFAT pathways in epidermal keratinocytes. The outcomes suggest that CLE is a promising candidate that can protect against LPS-induced skin injury. 

## 2. Results

### 2.1. Analysis of Ethanolic Crataegus Laevigata Berry Extract

**Figure 1:**
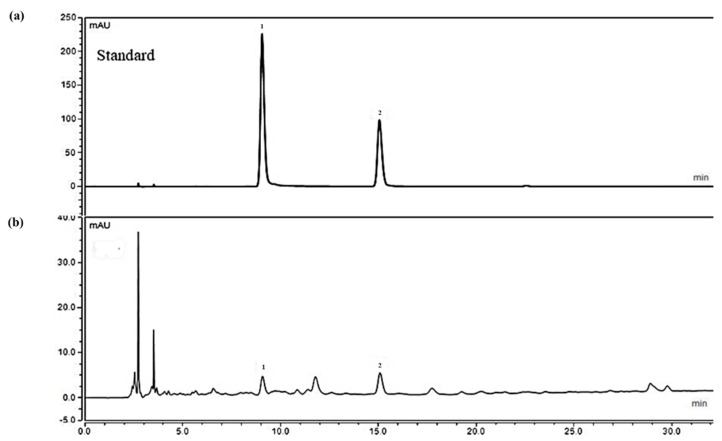
The HPLC (high-performance liquid chromatography) result of chlorogenic acid (peak 1) and (−)-epicatechin standards (peak 2) (**a**) and the contents of chlorogenic acid and (−)-epicatechin in *C. laevigata* berry extract (CLE) (**b**).

As shown in Figure 1b, chlorogenic acid and (−)-epicatechin peaks in CLE were detected at the time of 9.15 min and 15.21 min, respectively, by comparison with the retention times obtained from chlorogenic acid and (−)-epicatechin standards. The proportions of chlorogenic acid and (−)-epicatechin in CLE were 7.86 μg/g and 18.54 μg/g, respectively. 

### 2.2. Antioxidative Activity of CLE 

**Figure 2:**
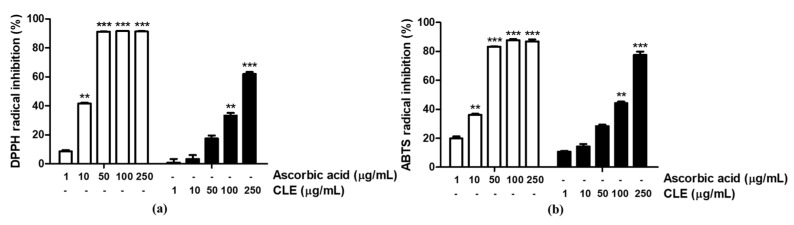
DPPH radical (**a**) and ABTS^•+^ cation (**b**) scavenging activity of *C. laevigata* berry extract (CLE). The radical scavenging effect was presented as a percentage of that measured in the control group. Data are presented as the mean ± SD. ** and *** indicate the significant within-group differences (** *p* < 0.01 and *** *p* < 0.001, respectively).

The free radical scavenging activity of both ascorbic acid (positive control) and CLE was dose-dependent. In the DPPH assay, the IC_50_ values for ascorbic acid and CLE were 11.15 µg/mL and 189.60 µg/mL, respectively (Figure 2a). Meanwhile, the ABTS^•+^ cation scavenging effect of ascorbic acid and CLE resulted in IC_50_ values of 16.15 µg/mL and 99.82 µg/mL, respectively (Figure 2b).

**Figure 3:**
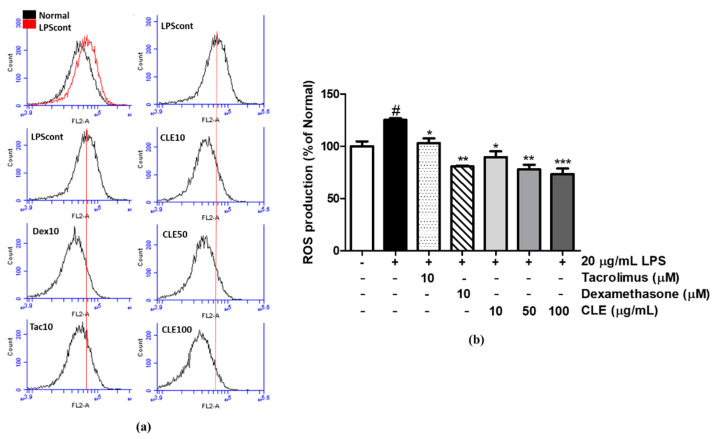
Levels of reactive oxygen species (ROS) in human keratinocytes (HaCaTs) after 24 h of treatment were determined by flow cytometry with DCFH-DA dye. The number of cells is plotted versus the dichlorofluorescein fluorescence detected by the FL-2 channel (**a**). The relative ROS production of cells is presented as histograms (**b**). Values are shown as mean ± SD. # and * indicate significant differences from the non-LPS-treated control and LPS-induced control groups, respectively. ^#^ *p* < 0.05 vs. the non-treated group. *, **, and ***p < 0.05, 0.01, 0.001 vs. the LPS-treated control, respectively.

To evaluate the anti-oxidative effect of CLE on LPS-stimulated HaCaTs, cells were stained with 30 µM DCFH-DA. ROS formation was significantly induced by LPS stimulation (up to 125.34%); however, CLE and the two positive controls of tacrolimus and dexamethasone decreased ROS production by 41.34% at 100 µg/mL, 17.80% at 10 µM, and 36.00% at 10 µM, respectively, compared with the LPS-induced control group (Figure 3).

### 2.3. Cell Viability and Chemokine mRNA Expression Levels in LPS-Treated and CLE-Treated HaCaTs

**Figure 4:**
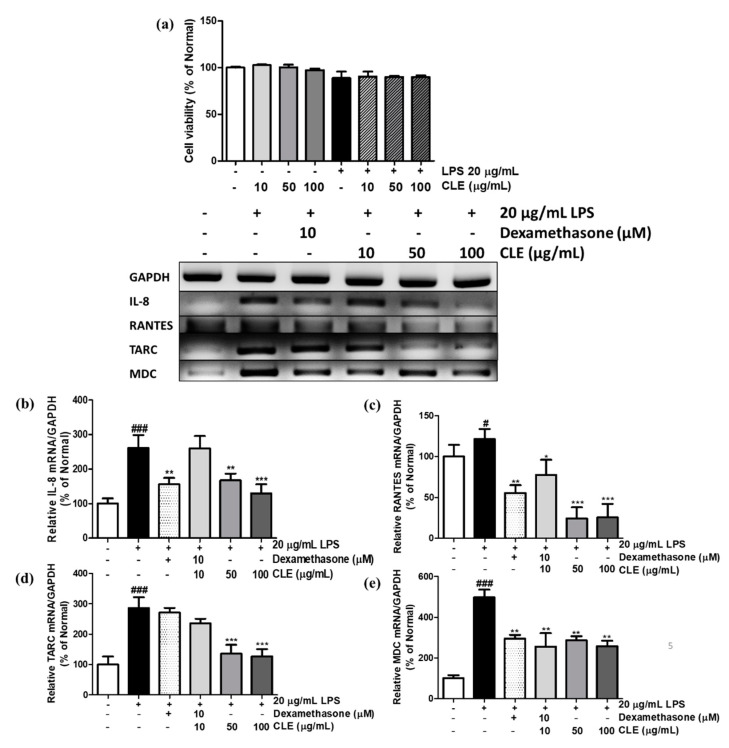
Effect of CLE on cell viability (**a**) and mRNA expression levels of IL-8 (**b**), RANTES (**c**), TARC (**d**), and MDC (**e**) under LPS stimulation. Values are shown as mean ± SD. # and * indicate significant differences from the non-LPS-treated control and LPS-induced control groups, respectively. # and ### *p* < 0.05 and 0.001 vs. the non-treated group, respectively. *, **, and *** *p* < 0.05, 0.01, and 0.001 vs. the LPS-treated control, respectively.

To evaluate the cytotoxicity of CLE on HaCaTs, cells were cultured in both non-treated and LPS-treated conditions. LPS reduced cell viability to 88.82%. CLE-treated keratinocytes did not exhibit marked cytotoxicity (Figure 4a). 

As shown in Figure 4b–e, treatment of keratinocytes with LPS upregulated the mRNA levels of interleukin 8 (IL-8), chemokine (C-C motif) ligand 5 (CCL5) (also known as RANTES), chemokine (C-C motif) ligand 17 (CCL17) (also known as TARC), and chemokine (C-C motif) ligand 22 (CCL22) (also known as MDC) by 161.55%, 21.27%, 185.86%, and 397.60%, respectively. Compared to LPS controls, treatment with dexamethasone showed significant effects in suppression of IL-8, RANTES, and MDC mRNA production by 40.34%, 54.28%, and 40.76%, respectively. However, under LPS induction, only CLE supplement at 50 and 100 µg/mL exhibited lower TARC mRNA production, which is reduced by 52.82% and 55.58%, respectively. CLE also presented remarkable inhibitory effects on mRNA expression of IL-8 by 50.42% (at 100 µg/mL). Notably, a low dose of CLE (10 µg/mL) exhibited RANTES and MDC inhibition by 36.08% and 48.19%, respectively (Figure 4c,e).

### 2.4. Regulatory Effect of CLE Treatment on MAPK Signaling Pathway and AP-1 Activation

**Figure 5:**
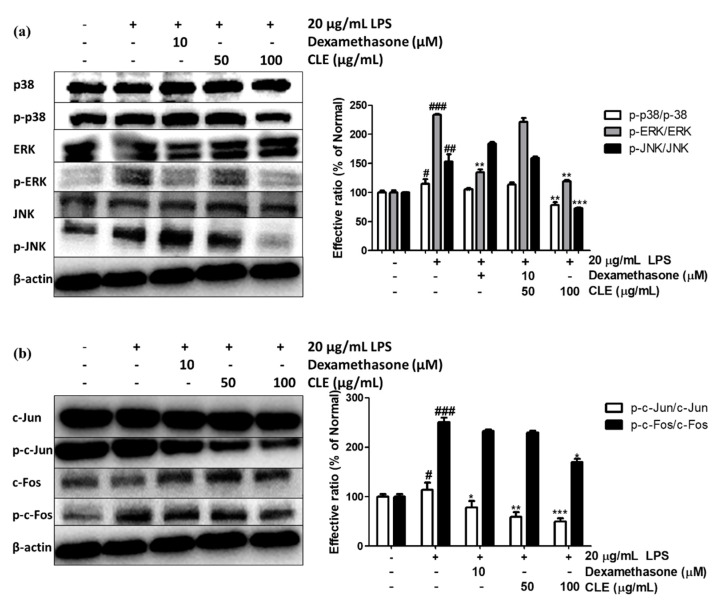
Effect of CLE on protein expression levels of phosphorylation of MAPKs (**a**) and AP-1 subunits c-Jun and c-Fos (**b**) under LPS stimulation. Values are shown as mean ± SD. # and * indicate significant differences from the non-LPS-treated control and LPS-induced control groups, respectively. #, ##, and ### *p* < 0.05, 0.01, and 0.001 vs. the non-treated group, respectively. *, **, and *** *p* < 0.05, 0.01, and 0.001 vs. the LPS-treated control, respectively.

As shown in Figure 5a, LPS-induced ROS formation triggered the phosphorylation of p38, ERK, and JNK, increasing the levels by 14.97%, 133.70%, and 153.06%, respectively. Importantly, pretreatment with CLE reversed these changes, suppressing the phosphorylation of these signaling molecules at both 50 and 100 μg/mL compared to the LPS control. At 100 µg/mL, CLE down-regulated the p-p38 expression by 31.86%, the p-ERK expression by 49.89%, and the p-JNK expression by 52.58%; dexamethasone at 10 µM only showed reduction in p-ERK expression by 42.38%.

MAPK is a key regulatory pathway controlling activation of AP-1, which consequently activates the promoter of inflammatory chemokine and cytokine genes and promotes mRNA expression. To study the mechanism underlying the effects of CLE on AP-1 family members, phosphorylations of c-Jun and c-Fos were recorded in HaCaT cells exposed to 20 µg/mL LPS. As shown in Figure 5b, although LPS stimulated phosphorylation of c-Jun and c-Fos by 14.25% and 150.83%, respectively, CLE treatment strongly suppressed expression of these proteins by 56.26% and 32.28%. The positive control dexamethasone did not downregulate p-c-Fos but did exhibit relative inhibition of p-c-Jun (31.33%).

### 2.5. Regulatory Effect of CLE Treatment on NFκB Signaling Pathways

**Figure 6:**
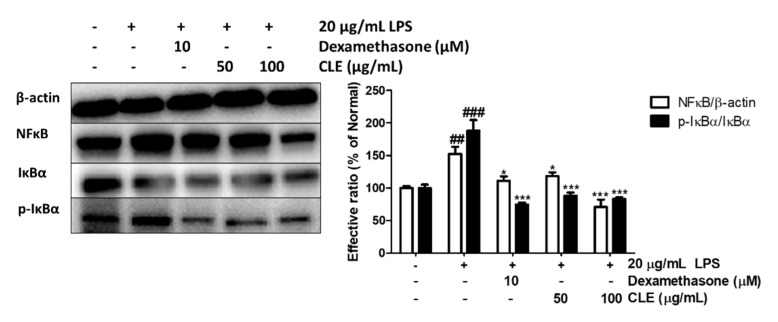
Effect of CLE on protein expression levels of NFκB signaling pathway components under LPS stimulation. Values are shown as mean ± SD. # and * indicate significant differences from the non-LPS-treated control and LPS-induced control groups, respectively. ## and ### *p* < 0.01 and 0.001 vs. the non-treated group, respectively. *, **, and *** *p* < 0.05, 0.01, and 0.001 vs. the LPS-treated control, respectively.

As shown in Figure 6, LPS treatment stimulated NF-κB expression and the phosphorylated form of inhibitor of kappa B alpha (IκBα) by 52.33% and 87.95%, respectively. Nevertheless, CLE pretreatment at concentrations of 50 and 100 μg/mL effectively reduced NF-κB and p-IκBα levels by 22.10% and 53.37%, and 53.10% and 55.56%, respectively. Dexamethasone at 10 µM also markedly inhibited the NF-κB pathway, but the inhibitory effect on NF-κB expression was not as effective as that of CLE at 100 μg/mL.

### 2.6. Regulatory Effect of CLE Treatment on NFAT Signaling Pathways

**Figure 7:**
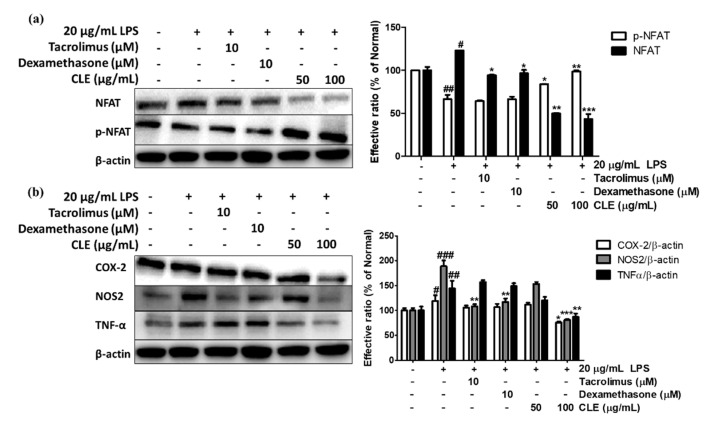
Effect of CLE on protein expression levels of NFAT signaling (**a**) and COX-2 (**b**) under LPS stimulation. Values are shown as mean ± SD. # and * indicate significant differences from the non-LPS-treated control and LPS-induced control groups, respectively. #, ##, and ### *p* < 0.05, 001, and 0.001 vs. the non-treated group, respectively. *, **, and *** *p* < 0.05, 0.01, and 0.001 vs. the LPS-treated control, respectively.

It was reported that NFAT plays a critical role in LPS-promoted inflammation. The LPS activation indirectly results in Ca^2+^ influx-dependent NFAT dephosphorylation. Cytoplasmic NFAT translocates to the nucleus and binds to the promoter regions of cytokine genes to promote transcription. As shown in Figure 7a, LPS exposure significantly promoted NFAT dephosphorylation and triggered a relative increase in NFAT expression. However, CLE prevented NFAT nuclear translocation by enhancing p-NFAT level. Incubation of cells with 100 µg/mL CLE led to a 48.47% increase in p-NFAT and reduced NFAT expression by 64.82% compared with the control group. Although calcineurin inhibitor tacrolimus or dexamethasone at 10 µM did not up-regulate p-NFAT, they exhibited relative inhibitory effects on NFAT of 23.52% and 21.63%, respectively.

CLE also exhibited suppression on some downstream targets of NFAT such as COX-2, NOS2, and TNF-α, which have essential roles in mediating the inflammatory reaction. LPS significantly increased COX-2, NOS2, and TNF-α by 19.12%, 89.07%, and 44.22%, respectively. CLE-treated cells (at 100 µg/mL) showed relatively low levels of these three proteins, whereas both tacrolimus and dexamethasone only markedly decreased NOS2 expression.

## 3. Discussion

In Europe and Eastern Asia, the genus *Crataegus* (Hawthorn berry) has been therapeutically applied for heart disease treatment [22,25], digestive support [30], blood circulation improvement [21,25], and pain relief [28]. Several studies have pointed out other pharmacological effects of hawthorn species on oxidative stress and the immune system, along with explanations of the molecular mechanism underlying these effects. Martin et al. reported that dry ethanolic extract from hawthorn leaves and flowers had a protective effect against inflammation-induced endothelial permeability by inhibiting Ca^2+^ influx in human umbilical vein endothelial cells (HUVECs) [31]. A study by Idris-Khodja et al. on aging-related endothelial dysfunction in rats showed COX-1 and COX-2 suppression of ethanol extract from hawthorn leaves, resulting in reduction of ROS production [32]. In addition, *C. laevigata* showed inhibition of cytokine TNF-α release from LPS-induced neutrophils [33]. Although there is an increasing number of reports about health benefits of hawthorn species, the protective effects of *C. laevigata* on oxidative stress and inflammation in relation to skin disease have not been elucidated.

In the current study, 70% ethanol *C. laevigata* berry extract (CLE) showed high contents of the active components of chlorogenic acid and (−)-epicatechin, widely known for their free radical capacity [28,34,35] and anti-inflammatory effects [36,37]. In particular, chlorogenic acid showed protective effects against dextran sodium sulfate-induced ulcerative colitis in mice through its inhibitory action on mitogen-activated protein kinase (MAPK) pathway [38]. Moreover, chlorogenic acid was reported to down-regulate the nuclear factor kappa-B (NF-κB) pathway and nuclear factor of activated T-Cells c1 (NFATc1) expression in osteoclast [39]. In our previous study, chlorogenic acid was also confirmed to prevent the nuclear localization of NFAT by promoting phosphorylated cytosol NFAT expression in UVB-radiated dermal fibroblasts [40]. In addition, Seo et al. demonstrated that the active compound (−)-epicatechin was able to reduce CC chemokines’ protein expression including TARC, RANTES, and MDC in LPS-treated RAW 264.7 macrophages and TNFα/INFγ-induced HaCaT keratinocytes [41]. Here, we hypothesized that CLE, which has both chlorogenic acid and (−)-epicatechin as the active components, possesses protective ability against LPS-induced oxidative stress during the inflammatory response in human keratinocytes. As expected, our results demonstrated that CLE protected human keratinocytes from LPS induction by suppressing formation of ROS involved in inactivation of MAPK/AP-1 and NF-κB nuclear translocation, resulting in down-regulation of chemokine and cytokine mRNA expression. Especially, we also detected the blocking effect of CLE on de-phosphorylation of transcriptional factor NFAT, inhibiting inflammatory factors such as COX-2, iNOS, and TNF-α secretion; this suggests that CLE protects against LPS-induced oxidative stress and inflammation.

Lipopolysaccharide (LPS) is the essential component in the outer layer of the bacterial membrane. Under skin inflammatory conditions induced by bacterial infection, LPS mediates ROS and indirectly induces secretion of a variety of cytokines and chemokines. Keratinocytes account for 90% of cell types in the epidermis, which is the outermost protective barrier of immune defense [42]. One of their functions is activation of dendritic cells to promote pro-inflammatory cytokine production of naive T helper cells [43]. In particular, keratinocyte-derived TARC, RANTES, and MDC have a major role in initiation of the inflammatory response [44,45]. Additionally, IL-8 is a cytokine that triggers chemotaxis in neutrophils and granulocytes, which are subsequently delivered toward the infected site [46]. In the current research, CLE dose-dependently suppressed both ROS formation and mRNA production of pro-inflammatory cytokine IL-8 and chemokines RANTES, TARC, and MDC in LPS-induced HaCaTs. Therefore, it can be concluded that CLE prevents overexpression of inflammatory mediators by inhibiting excessive ROS synthesis. 

In this research, the protein levels of early signaling molecules of LPS-induced keratinocytes were investigated. The MAPK family includes extracellular signal-regulated kinase (ERK), c-Jun N-terminal kinase (JNK), and p38 mitogen-activated protein kinase (p38), which regulate various physiological processes [47]. As immune activators such as LPS trigger ROS formation, MAPKs experience signaling transduction that leads to phosphorylation of p-p38, p-ERK, and p-JNK. This induction causes nuclear localization of heterodimer AP-1, which is made up of subunits c-Fos and c-Jun that bind to promoter regions of pro-inflammatory cytokines and chemokines for initiation of transcription [48,49]. Here, we found that CLE dose-dependently reduced protein levels of phosphorylated forms of both MAPK and AP-1 (Figure 5), which explains the decrease in mRNA of chemokines and cytokines in CLE-treated keratinocytes (Figure 4b–e). 

In addition, transcription factor NF-κB is widely investigated for its regulatory effect on activation of immune defense and inflammatory responses [50]. The inactivated form of NF-κB is attached with inhibitor of kappa B (IκB) in the cytoplasm [51]. Upon induction of LPS, the IκB-kinase (IKK) complex is triggered to phosphorylates IκB, resulting in proteasomal degradation of IκB [50]. The released NF-κB is translocated to the nucleus where it indirectly initiates the mRNA synthesis of target genes such as IL-8, TARC, and RANTES [52]. Our results revealed that CLE significantly prevented LPS-induced activated NF-κB by suppressing IκB phosphorylation.

NFAT protein is maintained in an inactivated state and predominantly found in the cytoplasm, where it can be converted to dephosphorylated form by the changes of intracellular calcium concentration and subsequently translocated to the nucleus [53]. Several reports state that LPS-triggered release of proinflammatory molecules resulting from overexpression of NFAT-regulated genes plays critical roles in the inflammatory process [18,54]. In addition, NFAT is involved in TSLP production in keratinocytes, which leads to psoriasis, eczema, and atopic dermatitis [53,55]. As serious adverse effects of topical steroid therapy have been widely reported, the alternative drug tacrolimus, a calcineurin inhibitor, is applied in chronic inflammatory treatment. However, tacrolimus produces side effects such as nausea, diarrhea, and constipation. Therefore, there is increasing demand for safer natural products for skin inflammatory therapy. The current research showed that CLE strongly down-regulated LPS-induced dephosphorylated NFAT by positively interfering with the p-NFAT protein level. Moreover, CLE significantly reduced COX-2, NOS2, and TNF-α secretion, which is related to downstream regulation of NFAT [17,54]. Thus, CLE might be an alternative and effective agent to dexamethasone or tacrolimus to treat skin diseases.

## 4. Materials and Methods

### 4.1. Chemicals

DMEM (Dulbecco′s Modified Eagle′s Medium) and fetal bovine serum (FBS) were obtained from Hyclone (HyClone Laboratories Inc., Logan, UT, USA). Antibiotic-Antimycotic was purchased from Gibco (Grand Island, NY, USA). HPLC standard compounds (chlorogenic acid and (−)-epicatechin), 3-(4,5-Dimethylthiazol-2-yl)-2, 5-diphenyltetrazolium bromide (MTT), and dimethyl sulfate (DMSO) were purchased from Sigma-Aldrich (St. Louis, MO, USA). Organic solvents (chloroform, isopropanol, methanol, and ethanol) were purchased from Samchun Chemical (Seoul, Korea) and Daejung Chemical & Metal (Siheung, Korea). Inorganic salts were purchased from Sigma-Aldrich. Silica gel was purchased from Merck (Kenilworth, NJ, USA).

### 4.2. Sample Preparation 

*Crataegus laevigata* (midland hawthorn) was purchased from Starwest Botanicals, Inc. (Sacramento, CA, US), and 300g of dried berries were extracted two times for 24 h at room temperature in 70% ethyl alcohol at a ratio of 1:8 (*w*/*v*). The extract was obtained by filtration (Whatman, USA), and the solvent was evaporated under vacuum.

### 4.3. HPLC Analysis

High-performance liquid chromatography (HPLC) was performed on a Dionex Chromelon TM chromatography data system with P580 and UVD100 detectors (Thermo Fisher Scientific Inc., Waltham, MA, USA). Analyzed sample (CLE) was prepared in 50% methanol at 5mg/mL. Ranges of diluted concentrations (3.125–100 µg/mL) of standard chlorogenic acid and (−)-epicatechin were dissolved in 50% methanol. Chromatographic separation was performed on a Waters 120 ODS-AP (250 × 4.6 mm^2^, 5-µm particle size). Elution was performed with a water/acetonitrile (3:1) gradient containing 1% formic acid. The gradient was linearly increased from 5% to 90% acetonitrile over 35 min. The injection volume was 10 µL and the flow rate was 1 mL/min. Analysis of chlorogenic acid and (−)-epicatechin in CLE was validated by using the external standard method, according to the International Conference on Harmonization [56] (see Supplementary Materials). Linear correlations (R^2^) of the calibration curves for each compound were 0.999. The limit of detections (LOD) and limit of quantifications (LOQ) were less than 0.028 µg/g and 0.074 µg/g, respectively (see Supplementary Materials). Thus, established HPLC method was suitable for the quantitative analysis. 

### 4.4. 1,1-Diphenyl-2-picrythydrazyl Radical Scavenging Activity

The scavenging effect of CLE on the free radical 1,1-diphenyl-2-picrythydrazyl (DPPH, PubChem CID: 2375032) was analyzed. A range of CLE concentrations (1, 10, 50, 100, and 250 µg/mL) was arranged on 96-well plates to measure the changes in optical density. DPPH radical solution was prepared at 0.2 mM in 100% methanol. Following addition of DPPH, the sample plate was covered with aluminum foil to prevent light-induced degradation, and the plate was incubated at 37 °C for 30 min. After incubation, the absorbance values were measured at 595 nm by a microplate reader (Molecular Devices FilterMax F5; San Francisco, CA, USA). The percentage of DPPH radical inhibition was determined using the following formula:

$$\%\;\mathrm{of}\;\mathrm{DPPH}\;\mathrm{radical}\;\mathrm{inhibition}=\frac{\left({\mathrm{OD}}_{0}-{\mathrm{OD}}_{x}\right)}{{\mathrm{OD}}_{0}}\times 100$$

where OD_0_ is absorbance of negative control; OD_x_ is absorbance of various tested CLE and ascorbic acid concentrations.

### 4.5. ABTS^•+^ Radical Scavenging Activity

The ability of antioxidant molecules to trap ABTS radical cation (ABTS^•+^, PubChem CID: 5464076) was detected. A solution of ABTS^•+^ was formed by the reaction of a 2.5 mM ABTS solution with 1 mM 2,2′-azobis(2-amidinopropane) dihydrochloride (AAPH) and 150 mM sodium chloride and by incubating the mixture at 70 °C for 30 min. Various concentrations of CLE (1, 10, 50, 100, and 250 µg/mL) were ordered on a 96-well plate to test for ABTS^•+^ radical scavenging activity. The contents were mixed well with prepared ABTS^•+^ radical cation solution and incubated at 37 °C for 10 min. Then the absorbance was determined at 405 nm. After incubation, the absorbance values were measured by a microplate reader (Molecular Devices FilterMax F5; San Francisco, CA, USA). The percentage of ABTS radical inhibition was determined using the following formula:

$$\%\;\mathrm{of}\;\mathrm{ABTS}\;\mathrm{radical}\;\mathrm{inhibition}=\frac{\left({\mathrm{OD}}_{0}-{\mathrm{OD}}_{x}\right)}{{\mathrm{OD}}_{0}}\times 100$$

where OD_0_ is absorbance of negative control; OD_x_ is absorbance of various tested CLE and ascorbic acid concentrations.

### 4.6. Cell Culture 

The HaCaT cell line originating from human keratinocytes was purchased from the Korea Cell Line Bank (Seoul, Korea). Cells were cultured in DMEM medium containing 10% FBS and 1% penicillin-streptomycin at 37 °C in a humidified atmosphere containing 5% CO_2_. The treatment was conducted after cells reached 70–80% confluence. HaCaT cells were pre-treated with either 10–100 μg/mL of the CLE or 10 μM of positive control (dexamethasone and tacrolimus) for 1 h. Next, cells were exposed to 20 µg/mL LPS for 24 h in order to stimulate inflammatory response.

### 4.7. Cell Viability

After treatment period, 1 mg/mL 3-(4,5-Dimethylthiazol-2-Yl)-2,5-diphenyltetrazolium bromide (MTT) was supplemented to the cell culture, and then incubated for 2–3 h. After medium were removed, formazan crystals were dissolved in DMSO solvent. The absorbance was recorded at 595 nm using a FilterMax F5 microplate reader.

### 4.8. ROS Measurement

After 24 h of LPS stimulation, keratinocytes were stained with 30 µM 2′7′-dichlorofluorescein diacetate (DCFH-DA; Sigma-Aldrich) for 30 min. Cells were homogenized in 1X PBS and then analyzed by flow cytometry (BD Accuri™ C6 Plus Flow Cytometer, BD Biosciences, Qume Drive, San Jose, CA, USA).

### 4.9. Reverse-Transcription Polymerase Reaction

RNA isolation was performed by using TRIzol (Invitrogen, Waltham, MA, USA), according to the manufacturer’s instructions. Synthesis of complementary DNA (cDNA) was conducted by reverse transcriptase and oligo-(dT)15 dimer (Bioneer Co., Daejeon, Korea). A polymerase chain reaction (PCR) was performed using a PCR premix (Bioneer Co.) in a Veriti Thermal Cycler (Applied Biosystems, Foster City, CA, USA). Information about primer sequences of TARC/CCL17, MDC/CCL22, RANTES, IL-6, and IL-8 are shown in Table 1. The separation of final PCR products was performed by agarose gel electrophoresis.

### 4.10. Western Blot Analysis

Cells were collected and lysed in RIPA buffer (Bio-Rad, Hercules, CA, USA). Protein calibration were performed using Bradford reagent (Bio-Rad, Hercules, CA, USA) with the standard curve of bovine serum albumin (BSA). Equal amounts of total protein were separated using SDS-polyacrylamide gel electrophoresis (SDS-PAGE) and then transferred to a nitrocellulose membrane (Amersham Pharmacia Biotech, Buckinghamshire, UK). Transfer membranes were blocked with either 5% skim milk or 5% BSA, and then incubated with a primary antibody (Santa Cruz Biotechnologies, Santa Cruz, CA, USA) overnight. After washing three times with 1X TBST, membranes were incubated with a secondary antibody (Cell Signaling, Danvers, MA, USA), protein levels were detected using electrochemiluminescence (ECL) detection reagents (Fujifilm, LAS-4000, Tokyo, Japan) and ImageMasterTM 17 2D Elite software version 3.1 (Amersham Pharmacia Biotech, Piscataway, NJ, USA).

### 4.11. Statistical Analysis

Data were shown as mean ± standard deviation (SD). One-way analysis of variance (ANOVA) was performed for a statistical comparison of treatments in GraphPad Prism 5.0 (GraphPad Software Inc., San Diego, CA, USA). *p* < 0.05 was considered statistically significant. # *p* < 0.05 was considered statistically significant compared to non-treated cells, while * *p* < 0.05 was considered statistically significant compared to LPS-induced keratinocytes.

## 5. Conclusions

To sum up, we revealed that *Crataegus laevigata* berry extract (CLE) effectively protected HaCaTs against LPS-induced oxidative during inflammatory response by decreasing ROS generation and expression of inflammatory mediators. The anti-oxidative and anti-inflammatory effects of CLE were owing to the existence of two active compounds, chlorogenic acid and (−)-epicatechin. The underlying mechanism of protective effect involved inhibition of the MAPKs/AP-1 and NFκB pathways. Moreover, CLE controlled the expression of COX-2, NOS2, and TNF-α by preventing dephosphorylation of transcriptional factor p-NFAT. The outcome of this study suggests that CLE is a potential natural material that can alleviate LPS-induced skin injury. Using CLE as an alternative treatment to dexamethasone or tacrolimus might be a safer and effective application for therapy of skin disorders. Nevertheless, further study is required to investigate the underlying mechanisms of CLE on the in vivo model.

## Figures and Tables

**Table 1:** Polymerase chain reaction (PCR) primers used in this experiment.

Gene	Sense	Antisense
TARC/CCL17	ATGGCCCCACTGAAGATGCT	TGAACACCAACGGTGGAGGT
MDC/CCL22	AGGACAGAGCATGGCTCGCCTACAGA	TAATGGCAGGGAGGTAGGGCTCCTGA
RANTES/CCL5	CCCCGTGCCCACATCAAGGAGTATTT	CGTCCAGCCTGGGGAAGGTTTTTGTA
IL-8	TCAGTGCATAAAGACATACTCC	TGGCATCTTCACTGATTCTTG

## Data Availability

The data presented in this study are available supplementary material Table S1: Regression equation, limit of detection (LOD), and limit of quantification (LOQ) during HPLC of chlorogenic acid and (−)-epicatechin standards.

## Supplementary Materials

The following are available online. Table S1: Regression equation, limit of detection (LOD), and limit of quantification (LOQ) during HPLC of chlorogenic acid and (−)-epicatechin standards.

## Sample Availability

Samples of the compounds are available from the authors.
