# Supplementary material for: Crataegus laevigata Suppresses LPS-Induced Oxidative Stress during Inflammatory Response in Human Keratinocytes by Regulating the MAPKs/AP-1, NFκB, and NFAT Signaling Pathways
Source: Molecules. 2021 Feb 6;26(4):869. doi: 10.3390/molecules26040869 (PMC7914440; doi:10.3390/molecules26040869)
Supplement: Supplementary file 1 [file molecules-26-00869-s001.pdf]

Table S1. Regression equation, limit of detection (LOD), and limit of quantification (LOQ) during HPLC of chlorogenic acid and (−)-epicatechin standards.

Compound	Regression equation	LOD [1] (μg/g)	LOQ [1] (μg/g)
Chlorogenic acid	y = 35.34x + 0.63	0.028	0.074
(−)-epicatechin	y = 12.35x − 5.18	0.022	0.062

$R^2 > 0.999$. [1] LOD and LOQ were calculated by the concentrations that generated peaks with signal-to-noise values (S/N) of 3 and 10, respectively.
